# Supplementary material for: A Comparative Analysis between Whole Chinese Yam and Peeled Chinese Yam: Their Hypolipidemic Effects via Modulation of Gut Microbiome in High-Fat Diet-Fed Mice
Source: Nutrients. 2024 Mar 27;16(7):977. doi: 10.3390/nu16070977 (PMC11013417; doi:10.3390/nu16070977)
Supplement: Supplementary file 1 [file nutrients-16-00977-s001.zip › nutrients-2922302-supplementary.pdf]

Table S1 Proximate nutritional composition of Chinese yam (g/100g dry weight basis)

Component	WY	PY
Carbohydrate	72.25 ± 0.32 ^{a}	74.50 ± 0.26 ^{a}
Protein	13.85 ± 0.06 ^{a}	13.57 ± 0.10 ^{a}
Total dietary fiber	7.75 ± 0.07 ^{a}	6.28 ±0.05 ^{b}
Insoluble dietary fiber	7.05 ± 0.05^{a}	6.24 ± 0.02 ^{b}
Soluble dietary fiber	0.70 ± 0.03 ^{a}	0.36 ±0.03 ^{b}
Fat	1.47 ± 0.01 ^{a}	1.49 ± 0.03 ^{a}
Ash	4.67 ± 0.04 ^{a}	4.15 ± 0.05 ^{a}

Means with different small letter superscripts in the same column are significantly different at $p < 0.05$. The letter "a" denotes the highest value.

Table S2 Composition of experimental diets [1]

Ingredient (g/kg)	NCD	HFD	Yam-based diets	
			HFD-WY	HFD-PY
Whole Chinese yam	0.00	0.00	250.00	0.00
Peeled Chinese yam	0.00	0.00	0.00	250.00
Casein, 80 Mesh	189.56	258.45	223.83	224.52
L-Cystine	2.84	3.88	3.88	3.88
Corn Starch	479.79	0.00	0.00	0.00
Maltodextrin 10	118.48	161.53	0.00	0.00
Sucrose	65.21	88.91	58.13	53.82
Cellulose, BW200	47.39	64.61	45.24	48.91
Soybean Oil	23.70	32.31	28.63	28.59
Lard	18.96	316.60	316.60	316.60
Mineral Mix	9.48	12.92	12.92	12.92
DiCalcium Phosphate	12.32	16.80	16.80	16.80
Calcium Carbonate	5.21	7.11	7.11	7.11
Potassium Citrate, 1 H2O	15.64	21.32	21.32	21.32
Vitamin Mix, V10001	9.48	12.92	12.92	12.92
Choline Bitartrate	1.90	2.58	2.58	2.58
FD&C Yellow Dye #5	0.04	0.00	0.00	0.00
FD&C Blue Dye #1	0.01	0.07	0.07	0.07
Total	1000	1000	1000	1000
% Energy and their source				
Protein	20	20	20	20
Carbohydrate	70	20	20	20
Fat	10	60	60	60
Total	100	100	100	100

[1] To equalize the carbohydrate, protein, fat, sucrose and fiber content (g/kg diet) of the HFD, HFD-WY, and HFD-PY, the maltodextrin, casein, soybean oil, sucrose, and cellulose were reduced in the HFD-WY and HFD-PY diets, respectively.

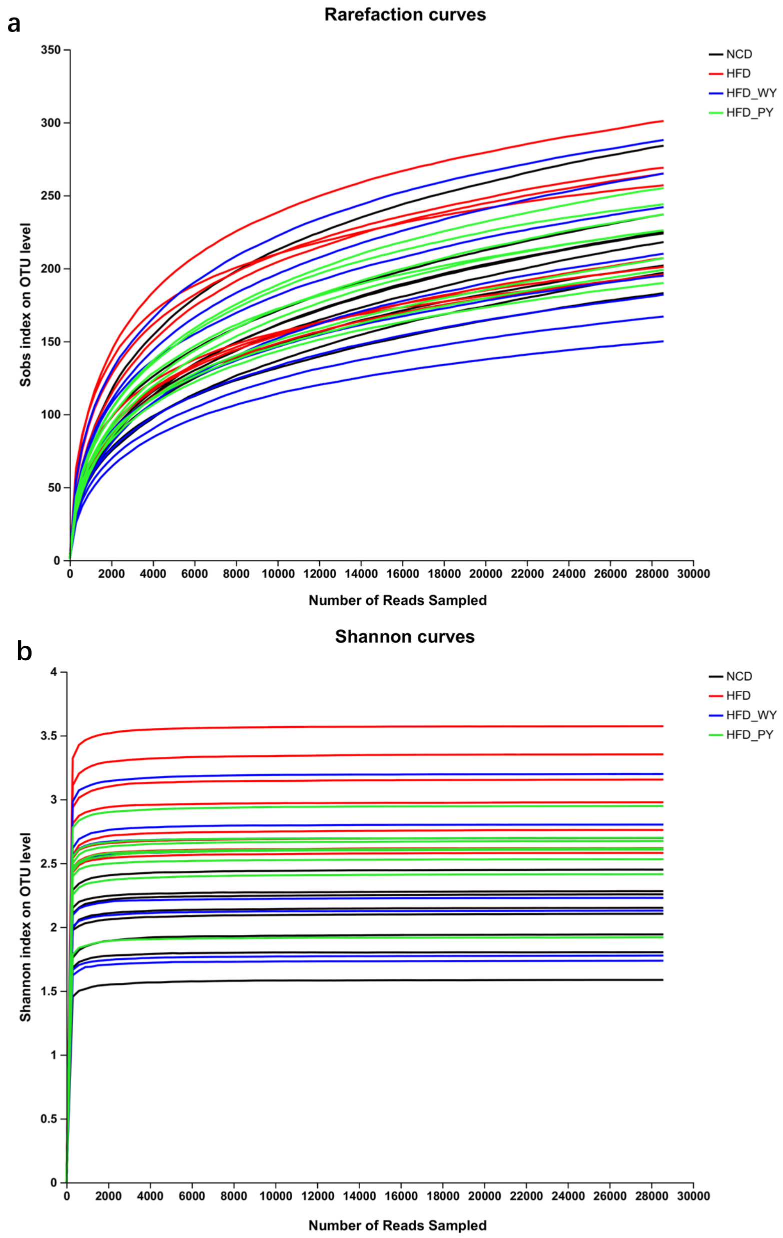

Figure S1 Alpha diversity analysis of samples by Rarefaction analysis (a) and Shannon index (b). Each bar represents one sample.
